# The Reasons for Discontinuation of Infliximab Treatment in Patients with Crohn's Disease: A Review of Practice at NHS Teaching Hospital

**DOI:** 10.5402/2011/672017

**Published:** 2011-10-26

**Authors:** Lukasz Z. Krupa, Hugh J. Kennedy, Crawford P. Jamieson, Nicola Fisher, Andrew R. Hart

**Affiliations:** ^1^Gastroenterology Department, Norfolk and Norwich University Hospital NHS Trust, Norwich NR4 7UY, UK; ^2^School of Medicine, Health Policy and Practice, University of East Anglia, Norwich NR4 7TJ, UK

## Abstract

*Introduction*. There is little information on the reasons for discontinuing infliximab treatment in patients with Crohn's disease. The aim of this study was to document these reasons to determine if any were preventable which would allow patients to continue the therapy. *Aims & Methods*. A review of the medical notes was conducted at the Norfolk and Norwich University Hospital on patients with Crohn's disease treated with infliximab between 2002–2008 to determine the reasons for stopping it. *Results*. A total of 65 patients were identified who had treatment with infliximab, of whom 23 (35.3%) had their therapy stopped. The reasons for discontinuation of infliximab in the 23 patients were: 47.8% side effects, 17.4% refractory disease, 13.0% achieved remission and did not receive long-term maintenance treatment, 4.34% pregnancy, 4.34% death, and unknown 13.0%. *Conclusions*. The main reasons for the discontinuation of infliximab were side effects rather than a lack of clinical response.

## 1. Introduction

Severe Crohn's disease is a debilitating illness which can have a devastating impact on the quality of life of patients who require life-long medication and frequently surgery. Patients with severe refractory disease not responding to initial drug treatment with 5-aminosalicylates and immunosuppressive agents may require biological therapies. These drugs, including infliximab, a monoclonal antibody, inhibit the actions of tumour necrosis factor alpha (TNF-*α*) whose concentration is increased in the mucosa of patients with Crohn's disease [1]. Infliximab currently induces and maintains clinical remission in 30%–40% of all patients who are commenced on therapy with intravenous infusions every eight weeks [2, 3]. Surprisingly, there is little information on why infliximab treatment is discontinued in patients and the subsequent clinical outcomes. The possibilities include lack of efficacy of the drug, anaphylaxis, severe sideeffects and patients' noncompliance. The purpose of this study was therefore to identify the reasons for discontinuation of infliximab and the clinical outcome in such patients, treated at a large teaching hospital. Identifying the reasons for discontinuation is important to determine if any are preventable or treatable, so that potentially more patients could benefit from biological therapies and achieve clinical remission. 

## 2. Methodology

The study was conducted in patients with Crohn's disease treated at the Norfolk & Norwich University Hospital NHS Trust, which serves a population of approximately half a million people in the East of England. Patients were identified from a database, in the Department of Gastroenterology, who were treated with infliximab between the years 2002–2008. The database also included those patients who had their treatment discontinued. Information was collected on factors which affect the clinical response to infliximab including smoking and the use of immunosuppressive drugs, namely, azathioprine and 6-mercaptopurine. The medical case notes of all patients who had their infliximab therapy discontinued were reviewed by a gastroenterologist to obtain information on demographic factors and the reasons for discontinuation (including lack of clinical response, side effects, social reasons, and noncompliance) and the long-term clinical outcomes.

## 3. Results

A total of 65 patients were treated with infliximab at the Norfolk and Norwich University Hospital between 2002 and 2008 of which 35.3% (*n* = 23) had their treatment discontinued. In the nonresponders the median age at first infusion was 35 years (range = 18–74 years), 65.2% were females, 60.7% of the patients (*n* = 14) were nonsmokers, 21.7% ex-smokers, and 17.4% continued to smoke. Crohn's disease was present at multiple sites in the gastrointestinal tract in 78.3% (*n* = 18) patients and in only the terminal ileum in 21.7% (*n* = 5). All patients had had a trial of a thiopurine. The median number of infusions before treatment discontinuation was 3 infusions (range = 1–20 infusions). 

The reasons for discontinuation of infliximab were: 47.8% (*n* = 11) side effects, 17.4% (*n* = 4) refractory disease, 13.0% (*n* = 3) achieved remission and did not receive maintenance treatment (as recommended by initial NICE guidance) [3], 4.5% (*n* = 1) pregnancy, 4.34% (*n* = 1) death (Pneumocystis carinii pneumonia), and 13.0% (*n* = 3) were unknown. In the subgroup who had side effects, these were (expressed as % of number with any side effect): 36.4% (*n* = 4) with severe anaphylactic reactions, 27.2%  (*n* = 3) arthralgia, 9.0% (*n* = 1) acute left ventricular failure, 9.0% (*n* = 1) cholestasis, 9.0% (*n* = 1) hypertension with a systolic blood pressure over 200 mmHg, and 9.0% (*n* = 1) recurrent headaches. Among the subgroup with severe anaphylaxis (*n* = 4), one patient developed shortness of breath, sweating, and rash during an infusion 2 years after the commencement on infliximab. The second patient developed wheeze and became hypotensive after the second infusion. Another patient experienced severe headaches, rash, and facial swelling nine days after the second infusion. The fourth patient had paresthesia and diffused rash while receiving the first infusion. Overall the side effects led to discontinuation of treatment in 11 of the 65 (16.9%) treated with infliximab. 

The final outcomes in the 23 patients in whom infliximab was discontinued were as follows: 11 patients (48%) underwent surgery, 3 patients had a total colectomy, 2 patients loop ileostomy, 1 proctectomy, 1 small bowel resection, 1 patient had terminal ileal resection, 1 ileocaecal resection, 1 patient drainage of ischiorectal abscess, and 1 repair of colovaginal fistula and subsequent hysterectomy. A further 22% of the 23 patients were maintained without further biological agents, 16% (*n* = 4) the outcome was unknown, 8% (*n* = 2) were commenced on adalimumab, and 4% (*n* = 1) died.

## 4. Discussion

Long-term infliximab treatment is beneficial for approximately 60 percent of patients who initially respond, leading to avoidance of hospital admissions and surgery [4]. In our study, a third of patients commenced on infliximab had their treatment discontinued, and the main reasons for this were side effects, particularly anaphylactic reactions and arthralgia. This emphasizes the importance of monitoring patients for this effect even if prophylaxis is given. More research is needed to document the prevalence and treatment of the arthropathy associated with biological therapies. Three patients had the treatment stopped after achieving remission, which is not current practice, but was the standard approach at the time. However, NICE in their final current approval determination document state that biological therapies should be given as a planned course or until treatment failure, or 12 months after the start of the treatment, whichever is the sooner [5]. Treatment with biological therapies should be continued if there is clear evidence of ongoing active disease as determined by symptoms, markers of inflammation, and possibly endoscopy. This guidance has been suggested in view of lack of information on the long-term efficacy of biological therapies and the severe but rare complications of the treatment including hepatosplenic lymphoma and demyelination. Our work showed that those who cannot receive long-term therapy need careful monitoring as approximately half of them will require surgery later on. Furthermore, clinicians are becoming more familiar with prescribing alternative biological therapy if the first fails. The numbers studied were relatively small but gave an overview of the potential limitations of infliximab. The reasons for discontinuation of biological therapies have been assessed in other centres. A large observational, single-centre cohort study from Leuven University Hospital, Belgium included 614 patients with Crohn's disease who were treated with infliximab. In 11% (70/614) of patients, the treatment was discontinued because of side effects after a median of five infusions. These included acute infusion reactions in 2.4% (15/614) of patients and delayed infusion reactions in 5.3% (33/614) of patients. Serious infections were diagnosed in 0.8% (5/614) of patients including herpes zoster, abdominal tuberculosis, and Aspergillus infection. Three patients had to stop infliximab because of neurological complications (one patient with possible neuritis optica, one patient with a diagnosed central demyelinating lesion, and one patient with extensive multiple sclerosis-like neurological symptoms) [4]. 

In summary a third of patients in our study were not able to continue long-term treatment with infliximab. More patients may be able to continue if side effects of treatment can be better understood, prevented, and managed. Long-term followup of those whose treatment was discontinued is important as many may need surgery or an alternative biological therapy.
